# Fault tolerance in computational grids: perspectives, challenges, and issues

**DOI:** 10.1186/s40064-016-3669-0

**Published:** 2016-11-18

**Authors:** Sajjad Haider, Babar Nazir

**Affiliations:** 1 Department of Computer Science, Shaheed Zulfiqar Ali Bhutto Institute of Science & Technology (SZABIST), H-8, Islamabad, Pakistan; 2 Department of Computer Science, National University of Modern Languages (NUML), H-9, Islamabad, Pakistan; 3 Department of Computer Science, COMSATS Institute of Information Technology, University Road, Tobe Camp, Abbottabad, 22060 Pakistan

**Keywords:** Fault identification, Fault tolerance, Fault classification, Computational grid, Distributed computing

## Abstract

Computational grids are established with the intention of providing shared access to hardware and software based resources with special reference to increased computational capabilities. Fault tolerance is one of the most important issues faced by the computational grids. The main contribution of this survey is the creation of an extended classification of problems that incur in the computational grid environments. The proposed classification will help researchers, developers, and maintainers of grids to understand the types of issues to be anticipated. Moreover, different types of problems, such as omission, interaction, and timing related have been identified that need to be handled on various layers of the computational grid. In this survey, an analysis and examination is also performed pertaining to the fault tolerance and fault detection mechanisms. Our conclusion is that a dependable and reliable grid can only be established when more emphasis is on fault identification. Moreover, our survey reveals that adaptive and intelligent fault identification, and tolerance techniques can improve the dependability of grid working environments.

## Background

Grid computing is an extension of distributed computing environment where geographically distributed resources are shared, selected, and aggregated based on the availability, performance, and capability (Guimaraes et al. 2013). From the cluster computing point of view, a grid is a collection of clusters that is “grid is a cluster of clusters” (Haider 2007). Distributed computing consists of three major paradigms, namely: (a) cluster, (b) grid, and (c) cloud (Valentini et al. 2013; Hussain et al. 2013).

As the nodes and resources are dynamically added in distributed systems like grids and clouds, different types of uncertainties start creeping and chances of resource failures increase. According to in Moon and Youn (2015) 70–75% resources have failure rates of around 20 and 40% in workload archives such as DEUB, UCB and SDSC (Kondo et al. 2010). Furthermore, their application level traces reveal that most of their resources have more failure probabilities which further cause issues related to performance of scheduling and unavailability of resources (Kondo et al. 2010; Li et al. 2006). In many organizations, there are underutilized computing resources that can be effectively used by making them part of the grid. Desktop machines in organizations are busy less than 5% of the time (Viktors 2002). Servers available in organizations are un utilized to the full potential. For such scenarios, grid computing provides a paradigm for making use of such underutilized or unused idle resources in a better way to increase the efficiency of resource utilization. IBM has defined grid as Selic (2004). “Grid is a collection of distributed computing resources available over a local or wide area network that appears to an end user or application as one large virtual computing system. The grid’s vision is to create virtual dynamic organizations through secure, coordinated resource sharing among individuals, institutions, and resources. Grid computing is an approach to distributed computing that spans not only locations but also organizations, machine architectures, and software boundaries to provide unlimited power, collaboration, and information access to everyone connected to a grid”. Grid computing focuses on large scale resource sharing (Foster et al. 2001) where resources are distributed geographically in various administrative domains (Buyya and Murshed 2002; Yu and Buyya 2005).

Fault tolerance is a capability developed in the system to perform functions correctly even in the presence of faults. Taking fault tolerance into consideration would result in increased dependability of a grid system (Selic 2004). An important assumption in understanding fault tolerance is to know about the correct behavior of a system. A failure is encountered when a system moves away from an expected behavior. The cause of the failure is called error that ultimately depicts some sort of fault or defect in the system. More specifically, the fault is the real cause of a failure and error is merely an indication or sign of a fault. Multiple errors could be due to a fault, and even a single error could be the cause of multiple failures (Selic 2004).

Computational grids offer the constructs of large-scale applications, but the execution of the jobs are exposed to various types of failures. Resources can join or leave a grid dynamically. Therefore, dependability related issues, such as availability and reliability must be considered by the grid resource managers and job schedulers (Zadahmad Jafarlou and al 2012). A survey (Hwang and Kesselman 2003) regarding the problems expected in grids identifies, how job execution in a scalable and heterogeneous environment, such as a grid is a critical issue due to the likelihood of a wide range of failures. Grid applications are multi-tasked applications that require scalable, heterogeneous, and distributed environments for execution. Therefore, failure identification and failure handling techniques in such environments become application specific. If a job, whose results are expected within specific time intervals, fails to produce results within the time, then such a scenario is referred to as “timing related failure” (Siva Sathya and Syam Babu 2010; Garg and Singh 2011). Similarly, an application fails due to the difference in the variant versions of the grid middleware would be a “versioning fault” (Haider 2007). Another example is when an application attempts to write data on a hard disk, but cannot find the available space on the hard disk to perform the operation. As can be realized, there could be many cases where failures are expected to be encountered. The usage and implementation of grid will result in highlighting the significance of fault tolerance and the allied issues (Latchoumy and Khader 2012). Moreover, fault tolerance also plays a key role to ensure serviceability in cloud computing (Sun et al. 2013). To handle fault tolerance in cloud environments, Sun et al. (2013) have proposed a dynamic adaptive fault tolerant strategy.

Failure probability in grid computing environments is potentially higher due to its heterogeneous nature as compared to other conventional parallel computing environments (Nazir et al. 2012). Therefore, it is critical to perform beforehand measures to address the expected or even unexpected problems. Fault tolerance in grid environments can be divided into two major categories, namely: (a) fault tolerance using pro-active approaches and (b) post-active approaches (Garg and Singh 2011; Ganga et al. 2012). Pro-active fault tolerant approaches consider failures proactively before scheduling jobs on grid resources. Fault prediction and fault forecasting techniques are used in designing a proactive fault tolerant approach (Haider 2007; Haider and Ansari 2012). Proactive fault tolerance is relatively diffcult to implement as compared to reactive or post-active fault tolerant approaches (Zhang et al. 2009). Proactive fault tolerance approaches require different types of faults related knowledge with respect to the future (Haider and Ansari 2012). In the literature, most of the work regarding fault tolerance is based on post-active approaches rather than the pro-active approaches (Garg and Singh 2011; Haider and Ansari 2012). On the other hand, a post-active fault tolerant technique reacts or activates after the encountered failures. Reactive or post-active techniques uses fault identification techniques before responding to the occurred faults and only the identified faults can be tolerated (Haider et al. 2007). For example, if a network failure has occurred and a grid node is not responding due to the network failure, then a response to such a state could be in the form of a retry or replication (Haider et al. 2011). Here, retry or replication is the fault tolerant technique that will be applied after an identified problem, such as a network failure.

The major contributions of this work are as follows:

1. In this survey, a taxonomy of dependable grid computing is presented that identifies recent challenges and threats in grid computing. The presented taxonomy is an extension of Avizienis et al. (2004); however, our dependability taxonomy provides a rigorous and a more recent review. Moreover, additional challenges are discussed along with possible solutions that can be used to address such challenges. Similarly, threats to grid computing are discussed in more detail.
2. This paper presents a comprehensive survey on the types of errors, failures, and faults that are encountered in various grid computing environments. Nearly all of known types of risks that could be encountered in the grid environment are reviewed.
3. Lastly, based on the rigorous literature review, this survey identifies open research issues that need the attention of the research community to have more efficient solutions to a broad, complex, and challenging area of fault tolerance in the computational grids.

## Challenges in grid dependability

### Existing surveys

**Fig. 1:**
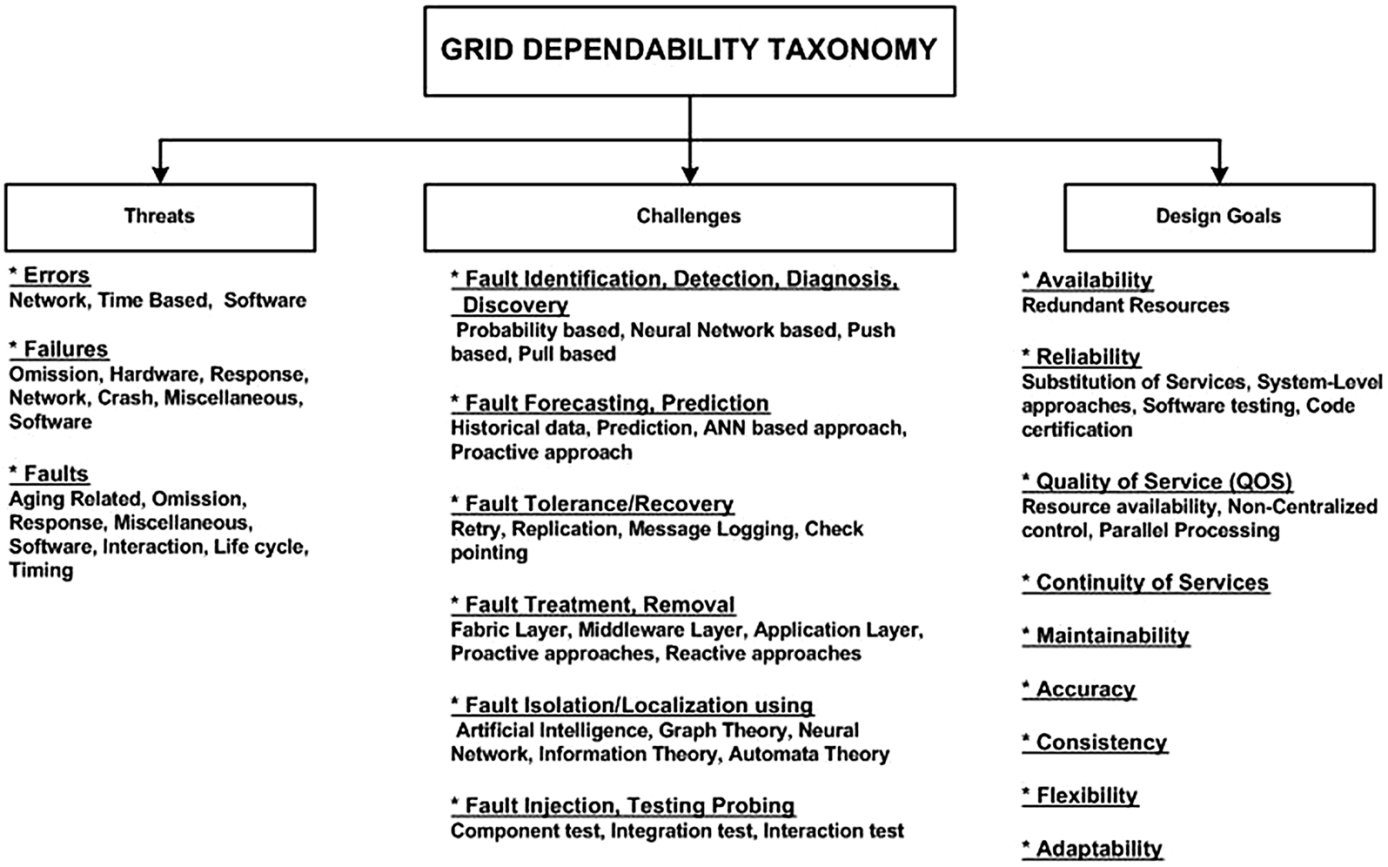
Grid dependability taxonomy

Many of the existing surveys on the dependability and security of computational grids are more focused on the computing systems in general, and do not pay more attention towards grid and distributed systems (Avizienis et al. 2004). Some of the surveys address fault tolerance in grid computing, but do not discuss in detail the types of threats and challenges (Latchoumy and Khader 2011). Some of the surveys focus more on the software side and ignore other areas, such as handling of the hardware based faults and their impact (Garg et al. 1998; Vaidyanathan and Trivedi 2001). Our survey specifically discusses the dependability scenarios in grid computing and most of the challenges, threats, and attributes related to dependability along with the corresponding subtypes that are specified in Fig. 1.

To attain high levels of availability and reliability, the infrastructure of grid must be fault tolerant (Qureshi et al. 2011). Avizienis et al. (2004) presented a dependability taxonomy that has been extended by incorporating more factors extracted from the literature. Challenges in the grid dependability are the factors that encompass fault identification, fault prevention, fault avoidance, fault forecasting, fault tolerance/recovery, fault treatment, fault isolation/localization, fault removal, fault diagnosis, fault injection, fault discovery, and fault testing. Similarly, there are some threats to the dependability that exist in the form of errors, failures, and faults and the corresponding subtypes. The design goals of a dependable grid system are availability, quality of service (QOS), reliability, consistency, maintainability, accuracy, flexibility, adaptability, and security. Fault tolerance is a vital aspect of grid for achieving reliability, availability, and QOS (Malik et al. 2012). Fuijan et al. (2012) proposed a QOS constrained algorithm for resource scheduling in grid environments by associating the tasks with QOS dimensions and one of the associated dimension was reliability.

The main strengths of this survey are that an enhanced/extended taxonomy of dependable grid computing is established that discusses various types of threats and the corresponding sub-types in more detail. In this survey, we also discuss various types of challenges faced by the grid computing environments to strengthen the dependability. Large numbers of papers were selected for review and to the best of our knowledge, we have discussed almost all of the types of challenges and their types along with examples faced nowadays. Similarly, the design goals have also been identified that can lead us to more reliable, available, and secure grid environments. Previously identified and published research (Nazir et al. 2012; Haider and Ansari 2012; Haider et al. 2011; Qureshi et al. 2011; Malik et al. 2012; Nazir et al. 2009; Khan et al. 2010) regarding fault tolerance pertaining to different types of errors, failures, and faults and the corresponding subtypes are also part of this survey, which discloses a very wide range of problems expected in the grid computing environments.

### Fault identification, detection, and diagnosis

**Fig. 2:**
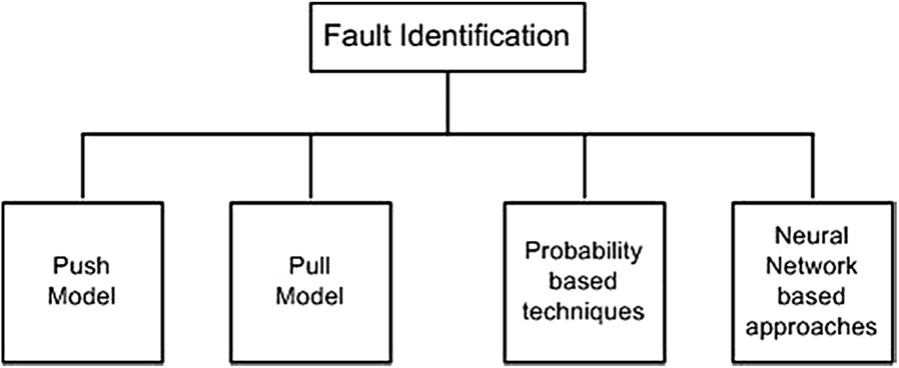
Techniques used in fault identification of computational grids

Fault identification, fault detection, and fault diagnosis are the techniques that are used to identify faults. Figure 2 depicts various techniques used in the fault identification of computational grids. A probabilistic and possibility risk assessment model for grid computing is proposed in Carlsson and Fuller (2010). A probabilistic resource allocation technique is applied by Shestak et al. (2012) considering the random failures in grid environments. Calado and da Costa (2006) used neural network based fault identification and diagnosis to claim that the fuzzy approach is most suitable for handling faults and achieving reliability in high performance computing environments. Charoenporwattana et al. (2008) used an artificial neural networks based approach to proactively avoid faults.

Faults are unavoidable in a complex distributed environment like grid that is scalable and heterogeneous. Diagnosing faults in such environments is a challenging task. A prompt detection and isolation mechanism of faults and failures lead to a reliable and robust environment. Automating fault diagnosis in large and complex distributed environments is critical (Sethi et al. 2004).

In a large-scale system many nodes performing tasks for applications related to computation, I/O and network communication etc. increase the probability of failures. The monitoring software dealing with nodes should be able to identify failures quickly (Massie et al. 2004). Massie et al. (2004) presented a monitoring system for HPC environments like clusters and grids with the name of Ganglia. Ganglia is based on hierarchical design which relies on multicast-based listen/announce protocol for monitoring sates within clusters. It further uses a tree of point-to-point connections of cluster nodes to merge clusters and combine their states.

Periodic device polling for monitoring information about the liveness of hardware or software has been used as fault detection and identification in distributed systems (Bheevgade and Patrikar 2008; Zhou et al. 2007; Bhagyashree et al. 2010). A technique used at software level for fault identification is known as “heartbeat” where a liveness message is produced by the device mentioning about its correct functioning and working (Ammendola et al. 2015), though it has a slight disadvantage of creating extra network traffic. In order to avoid the traffic problem a new trend is the use of Intelligent Platform Management Interface (IPMI) (Ammendola et al. 2015). An example of IMPI in high performance clusters is FTB-IPMI (Rajachandrasekar et al. 2012). Heartbeat and time-out method for handling the problem of unpredictable nodes in Map Reduce (MR) computations in hybrid computing environments has also been used by Tand et al. (2015).

### Fault localization and isolation

Fault localization is an important concept and is a part of fault management. Fault localization focuses on identification of the source of failure from a set of observed failure indications (Sethi et al. 2004). Fault localization is also referred to as fault isolation and alarm/event correlation. It is a set of observed fault indications that are analyzed to find the root cause of the problem (Katzela 1996).

Communication systems are constantly evolving and providing new capabilities, but on the other side they are also becoming more and more complex and the obligatory requirements on fault localization techniques have also changed. Fault localization in multifaceted and complex communication systems still remains an open research area (Sethi et al. 2004).

The proposed techniques in literature for fault localization and isolation are inspired from various areas of computer science, such as artificial intelligence, information theory, neural networks, graph theory, and automata theory. Such research areas can be used for identifying new dimensions in fault localization and isolation (Sethi et al. 2004).

### Fault injection and testing

Fault injection and testing are the techniques through which we asses the severity of the expected faults and the behaviors. In fault injection, faults are considered to be a valid case for a fault tolerant system, and are the techniques through which we can actually check the issues that can occur during the working of grid computing environments. Trodhandl and Weiss (2008) places fault injection methods into three main categories; (a) simulation-based fault injection (b) hardware-based fault injection, and (c) software-based fault injection. Brodie et al. (2003) claim that the problem determination and fault diagnosis can be performed using fault probing and testing for complex and scalable distributed systems.

Hsueh et al. (1997) emphasises on the importance of fault injection for evaluating the dependability of computer systems. Hardware and software based method exists for identifying the dependability through injecting faults in the systems. A case study of software based fault injection system for distributed systems is tested by Ghosh et al. (1997). It is recommended to apply fault injection techniques for identification of devastation in cases of failures. Severity and catastrophe of damage can be minimized through this type of proactive approach. Fault injection issues in distributed arena and especially in grid computing environments are a bit tricky as resources being tested are part of various geographical domains. Three way strategy, e.g. error based, coverage based and failure based is adopted by Ghosh et al. (1997) for implementation of fault injection.

- Error based strategy

This technique identifies error sources and then used techniques for injecting errors for each error category.

b. Coverage based strategy

Here coverage is measured with respect to code, interfaces and exceptions/errors codes.

c. Failure based strategy

Focuses on the behavior of system when other components fail and try to find whether faults are handled or not and whether the failure of one component affect the other one or not.

Cotroneo et al. (2013) is of view that fault injection methods mostly inject faults during experimentation phase and repeat the process various times. Advanced fault injection techniques start injection on encountering specific types of events in the system. Fault injection is a valid way for validating the fault tolerance technique (Fugini et al. 2009).

### Fault forecasting and prediction

Fault forecasting and prediction is a proactive way through which we can forecast or predict faults before they are actually observed, detected, and identified. A fault forecasting model for computer clusters was proposed by Haider and Ansari (2012), in which the forecasting and prediction of hardware faults is done on the basis of thermal signatures.

A java based neural network engine (JOONE) was utilized for fault predictions by Charoenpornwattana et al. (2008) and applicability of the ANN for fault prediction was discussed. Gurer et al. (1996) proposed an artificial intelligence based solution that incorporated an ANN based approach and case-based reasoning technique for fault handling in heterogeneous distributed environments. Prediction and forecasting of faults is an important method that can be used for improving the reliability of a system. Prediction and forecasting of faults can also be applied in proactive fault tolerance.

### Fault treatment and removal

Proactive and reactive fault tolerance techniques are placed in the fault treatment and removal category. The significant difference between them is that the proactive techniques anticipate and predict, while the reactive mechanisms react and respond.

**Fig. 3:**
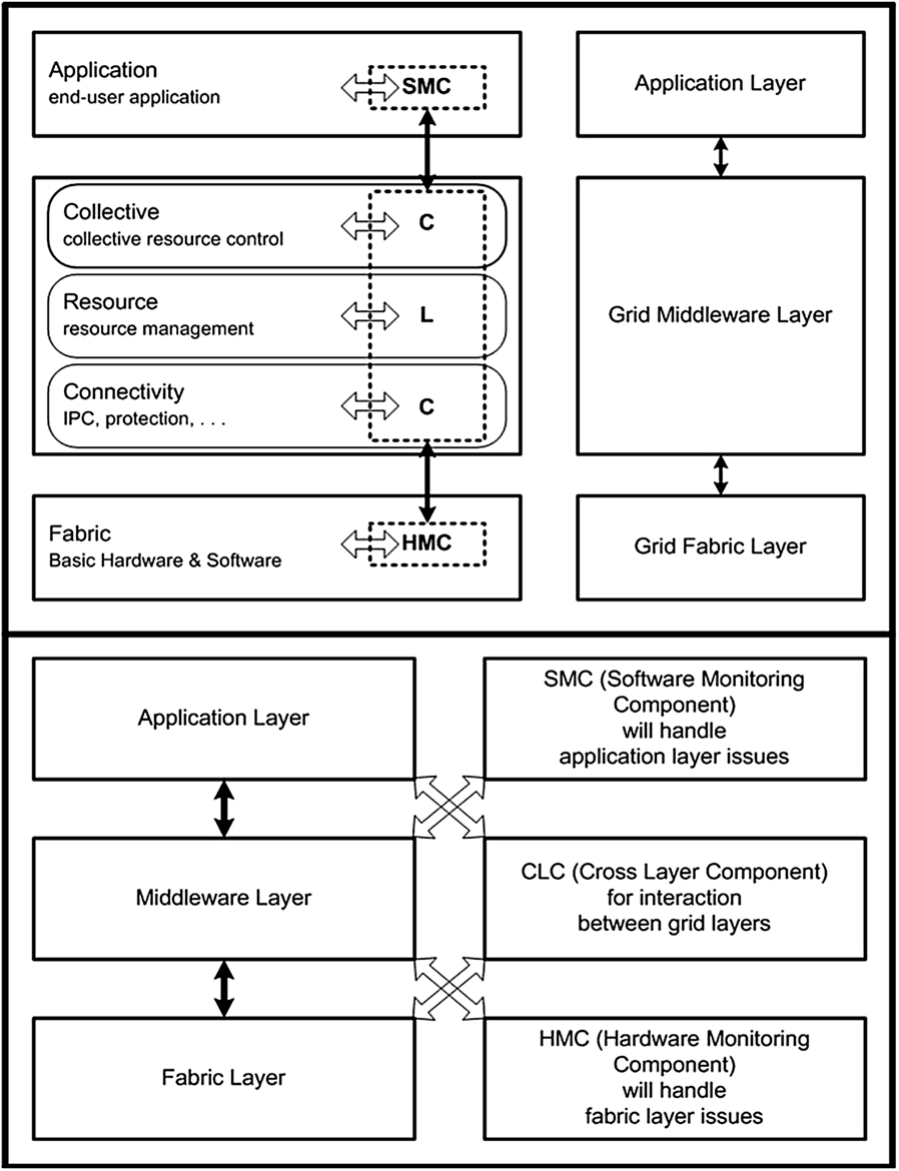
High level design diagram for component based proactive fault tolerant scheduling using cross layer design

A proactive fault tolerant scheduling approach is proposed by Haider et al. (2007) for handling the faults proactively in computational grids. The model uses various components at different layers of the grid that communicate with each other using a cross-layer design for calculating the overall reliability of the grid node. The use of cross-layered architecture is a relatively new concept that is applied in grid environments for handling faults proactively. Figure 3 shows a high level diagram of the proposed architecture. The architecture shows that at each layer of the grid, there is a component, and all of the components are communicating with each other using a cross-layer design.

The proposed model instead of using the traditional top-down/bottom-up approach of grid layers, uses a cross-layer communication model where a middle layer can communicate with the layers above and below it. Each layer of grid contains Hardware Monitoring Component (HMC), Software Monitoring Component (SMC) and Cross-Layer Component (CLC) for finding the most reliable nodes of the grid.

Hardware Monitoring Component (HMC) is designed to work at the fabric layer of grid. HMC is responsible for calculating Hardware Reliability Rating (HRR) of the machine/node and will rate each grid node as High, Medium or Low from HRR perspective. Factors considered for HRR are machine up time, remaining storage space of the node, OS service failures encountered by the node, network speed and connectivity time of the node with the network. A node which is up for long time and has large store space available and has high network connectivity speed etc. will be rated as HRR-High. Similarly a node having less storage space and slow network connectivity etc. will be rated as HRR-Medium. If a node is facing OS service failures and disconnecting with the network and has less up time then node will be rated HRR-Low.

Software Monitoring Component (SMC) works at application layer of grid and is responsible for calculating Software Reliability Rating (SRR) in the form of High, Medium and Low. A node is SRR-High when it has executed the task successfully, e.g. without encountering value faults, versioning faults, unhandled exceptions and unexpected inputs. Similarly SRR-Medium will be rated on encountering one or two faults, and SRR-Low will be assigned to a node upon encountering most of the faults, e.g. three or more faults.

Information generated about the reliability of the node in the form of High, Medium and Low with respect to Hardware and Software by HRR and SRR through HMC and SMC working at fabric and application layers of grid respectively is passed to Cross-Layer Component (CLC). CLC will overall rate the node as High, Medium or Low, depending on the rating received from HMC and SMC. For a node having HMC and SMC as High, the node is declared as highly reliable node. Similarly, if CLC receives information in the form of Medium then node is declared as Medium from reliability point of view and Low in case of information received as Low.

When grid scheduler selects nodes for execution of jobs, then only highly reliable nodes are selected as their reliability from hardware and software point of view has already been obtained.

Similarly, a framework for proactive fault tolerance is presented in Vallee et al. (2008) that uses a component based approach consisting of three components: (a) fault predictor, (b) policy daemon, and (c) fault tolerance daemon. Most (Kondo et al. 2010; Bahman arasteh et al. 2012; Hwang and Kesselman 2003; Garg and Singh 2011; Latchoumy and Khader 2011; Vaidyanathan and Trivedi 2001; Shestak et al. 2012) of the fault tolerant techniques discussed in literature use reactive techniques and solutions that employ preventive measures. When an application encounters a failure then instead of avoiding that failure, recovery technique are applied in order to handle the situation (Engelmann et al. 2009).

### Fault tolerance and recovery

Fault tolerance, recovery, and removal are solutions for the fault related problems in grid computing environments (Haider et al. 2011). Retry, replication, message logging, and check pointing are the fault tolerant techniques that are used in clustered and grid computing environments (Haider and Ansari 2012). Almost all of the fault tolerant solutions presented in the literature use the above mentioned techniques and we briefly discuss them below.

#### Retry

In retry, if a problem occurs in a distributed application, and due to that problem the application stops, then instead of finding the cause of the problem, we restart the application. Retry is considered to be the simplest failure recovery or fault tolerance technique. That is to say that, we hope that whatever were the causes of failures, the effect will not be encountered in the subsequent retries (Hwang and Kesselman 2003).

#### Replication

In replication, we run multiple copies or replicas of an application on different machines/nodes of the grid. The intention of running replicas on various machines/nodes is that if all of the machines fail and only a single machine out of those machines completes the job successfully, then the objective will be accomplished. The main idea of replication is to have replicas of a task run on different grid resources. As long as, not all of the replicated tasks crash, for example (due to host crash or host partition away from the grid client), then the task execution would succeed (Hwang and Kesselman 2003).

#### Message logging

Message logging is another technique used to handle faults in distributed systems. When an application executes, the nodes maintain the information about the execution of the application in the form of logs. If an issue is encountered, then the logs are used for an appropriate solution. In message logging, nodes log incoming messages to stabilize storage devices. After a failure, the message logs are used to compute a consistent global state. Algorithms that use the approach of message logging for fault tolerance are further classified into the following two categories: (Sistla and Welch 1989).

- Optimistic message logging

In optimistic message logging approach, a process starts execution before the completion of logging a message (with a hope that process will not encounter failure), but on encountering failure in such cases, chances are to have an orphan process. An orphan process will not be consistent with its associated process as it does not have the complete information about the associated process. Optimistic message logging approach creates orphan processes.

b. Pessimistic message logging

In pessimistic message logging approach there are no chances of orphan processes as a process does not proceed further unless it completely stores its state. Slight disadvantage in pessimistic approach is the time taken to store/log the complete message (Alvisi and Marzullo 1998).

#### Checkpointing

The most popular fault-tolerance mechanism is that of checkpointing. In this technique, we periodically save the state of the application on stable storage, usually a hard disk. After a crash, the application is restarted from the last checkpoint rather than from the beginning (Hussain et al. 2006). Checkpointing is a proficient way for developing a fault tolerant application. Bouguerra et al. (2013) have proposed a performance model through which checkpoint based scheduling problem has been expressed. Gokuldev and Valarmathi (2013) have discussed many types of checkpointing that include: (a) Full checkpointing, (b) Incremental checkpointing, (c) Coordinated checkpointing, (d) Uncoordinated checkpointing, (e) Kernel level checkpointing, (f) Application level checkpointing, and (g) User level checkpointing.

- Full checkpointing

Full checkpoint stores the complete state of the application to the local storage. Obvious drawback of this scheme is the time taken to save complete state and storage space required for storing the state.

b. Incremental checkpointing

Incremental checkpoint instead of storing the state of complete process, saves information of only the modified pages. Initially first checkpoint is the full checkpoint and the continuing checkpoints are stored on the basis of modified pages hence known as the incremental checkpoints. Incremental checkpoint technique is considered to be a reliable technique.

c. Coordinated checkpointing

In coordinated checkpointing, the protocols used for checkpointing generate reliable and steady checkpoints making overall recovery process to be simple. Through coordinated checkpointing technique a consistent global state can be maintained forcing participating processes to synchronize their checkpoints (Egwutuoha et al. 2013).

d. Uncoordinated checkpointing

In uncoordinated checkpointing every process takes its checkpoint independently and there is no coordination for checkpointing between processes. As there is no coordination between processes, there remains a chance for losing the complete computation and due to this very fact uncoordinated checkpointing technique is not used in practice (Egwutuoha et al. 2013).

e. Kernel level checkpointing

The process of checkpointing is included in the kernel and is transparent for the user so no modifications/changes are required in the program for the implementation of checkpointing. It is the responsibility of the kernel to manage recovery operations when the system restarts from a failure.

f. Application level checkpointing

In application level checkpointing it is the responsibility of the application to carry out all the checkpointing related issues. Checkpointing code and mechanism is part of the application and benefit of this technique is that checkpointing can be handled and controlled in a better way.

g. User level checkpointing

In this approach, user level library is linked with the application for checkpointing. Application code does not require any changes for incorporating checkpoint mechanism; however specific linking needs to be done between user level library and the application.

## Threats to grid dependability: errors, failures and faults

**Fig. 4:**
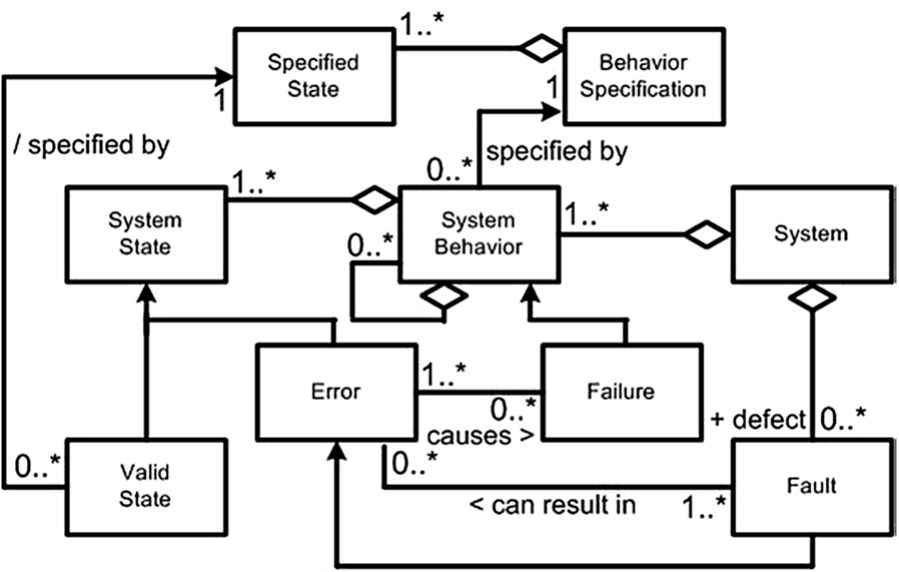
Relationship between errors, failures, and faults (Selic 2004)

An important assumption in understanding fault tolerance is to know about the correct behavior of a system. We generally say that a failure is encountered when a system moves away from the behavior for which it was designed. The reason behind that failure is called error, which ultimately depicts some sort of fault or defect in that system. This means that the fault is the actual and main reason behind a failure, and error is just an indication or sign of a fault. Multiple errors could be due to a fault, and even a single error could be the cause of multiple failures (Selic 2004). These concepts are shown in the unified modelling language (UML) class diagram, see Fig. 4.

In Fig. 4, we can see that fault and failures are not directly connected. Its reason is that fault or defect leads to error, whereas error leads to failure. Error will be produced due to the defect or fault of some hardware/software, due to which the task we wanted to perform will be halted resulting in failure. In simple words, faults results in errors that causes failures.

**Fig. 5:**
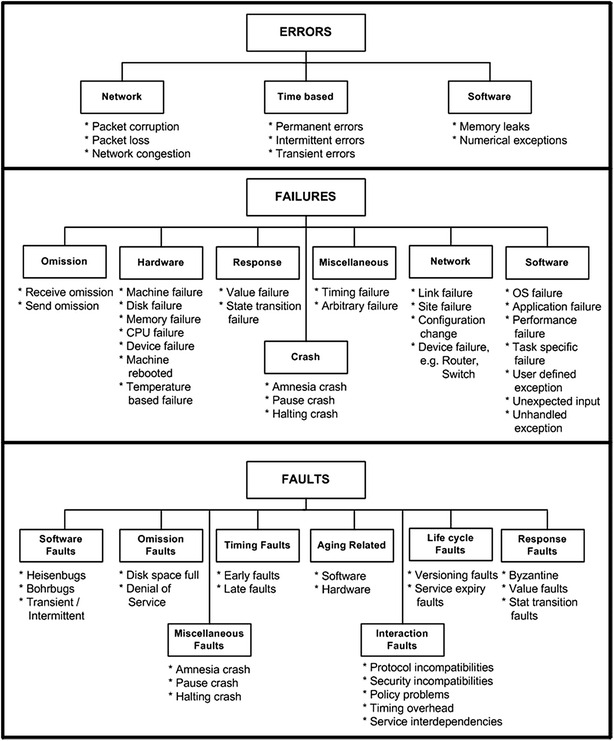
Extended classification of errors, failures, and faults (Haider and Ansari 2012; Haider et al. 2007; Haider et al. 2011)

Threats to grid dependability are established after a thorough literature survey. The classification of threats are specified with respect to various types of errors, failures and faults and the corresponding subtypes. In Fig. 5, we have identified various types of errors, failures, and faults, which we detail below.

### Error

Error can be observed and evaluated as a property of the state of the system. A system that starts facing the behavior against that system’s compliance and specifications is considered as an error. The following lists a few errors.

#### Network errors

In distributed environments, errors and failures related to nodes and or links are unavoidable and may cause a damaging effect on the performance of workflow based systems (Gu et al. 2013). Network errors can be in the form of packet corruption, packet loss, or network congestion (Siva Sathya and Syam Babu 2010).

- Packet corruption

In packet corruption, a packet gets corrupted during the transmission, when it moves from one node to the other. Noise can be a reason for packet corruption. Packet corruption can lead to further problems with respect to communication or change of information. The data that has to travel from the memory of source node to the main memory of target node has no protection. For example, if an error occurs after the validity of the data is verified by the network interface, but before calculating its CRC by the network, such type of error will go unnoticed and undetected (Balaji et al. 2012).

b. Packet loss

Packet loss is a problem in which a sent packet is lost during the transmission. If one or more packets of data do not reach the destination due to network errors, then such a problem is identified as a packet loss.

c. Network congestion

Network congestion is a problem that can be encountered due to low bandwidth. Diverting all of the traffic towards a single path can also create network congestion. More traffic or network load can also lead to network congestion. Network congestion creates delay in communication and in grid computing environment network congestion may affect QoS (Haider 2007).

#### Software errors

Numerical exceptions and memory leaks are identified as software errors.

- Memory leaks

Memory leaks are application specific problems in which an application uses a huge amount of memory and never releases that memory (Vaidyanathan and Trivedi 2001). It is not necessary that all memory errors originate from memory cells. There can be cases where memory contents are accurate and error occurs on the path from memory to processor (Balaji et al. 2012). Memory leak occurs when unneeded part of the memory is not released. According to Roohi Shabrin et al. (2006), memory leak is a problem in which a part of allocated memory can not be accessed, resulting in degradation of execution and performance of application. Application exhausts systems resources and ultimately program crashes due to the problem of memory leak.

b. Numerical exception

Applications require numerical computations during execution. An application that has not considered problems from the numerical conversions point of view is expected to generate numerical exceptions during execution. Unhandled exceptions that cause problems due to out of range produced values by applications are numerical exceptions.

#### Time based errors

Time based errors are generated due to the applications that do not complete the execution within a specified deadline, or the problems faced by the applications in different time intervals in a distributed environment. Transient, intermittent, and permanent errors are classified as time based errors (Arshad 2006). The probability of occurrence of a transient error is very less and they occur either very seldom or once in the life cycle of an application and then disappear. On the other hand, intermittent errors can be observed many times in an irregular fashion (Siva Sathya and Syam Babu 2010).

### Failure

The occurrence of failure is generally assumed on detecting some error in the system state (Haider et al. 2011). A failure can also be considered as a noticeable deviation from accepted specifications (Siva Sathya and Syam Babu 2010). Failures may be obvious in case of a detected error. Failure is actually observed when a deviated behavior is produced by the system instead of a normal or expected one. We have identified categories of failures that are, omission, hardware, response, network, software, crash, and miscellaneous failures, which we detail below.

#### Omission failures

Omission failures occurred and are observed when a server fails to react and respond to the incoming requests (Siva Sathya and Syam Babu 2010). Some observed omission failures are send omission and receive omission.

- Send omission

Send omission occurs when a server fails to send messages (Delporte-Gallet et al. 2005). A server that stops sending messages leads to serious issues, such as communication. A server that has stopped sending messages will be isolated in the network as it has lost the capability of communication, and grid is a network of computation that is useless with communication. In send omission failures a message that is sent by a process will not be placed into the communication channel (Delporte-Gallet et al. 2005).

b. Receive omission

In receive omission failures a message that has arrived at the communication channel will not be received by the algorithm of the process (Delporte-Gallet et al. 2005). Receive omission failure takes place when a server fails to receive messages. If a server stops receiving messages, then severe problem with respect to communication starts and the server and the connected nodes can not further proceed the business.

#### Hardware failure

Hardware failures are more obvious than many other types of failures. Although hardware failure is a general terminology there are many types of hardware failures, such as CPU failure, machine reboot, disk failure, memory failure, and device failure. Some hardware failures, such as disk, memory, and CPU are purely hardware based failures, but some hardware failures can be due to software, such as operating system. Egwutuoha (2014) has mentioned that hardware (processors, hard disks and memory etc.) are the reasons for more than 50% of the failures in High Performance Systems, and intensity of the workload affects the failure rate (Schroeder et al. 2010).

#### Response failure

Another category of failure is the response failure, where the grid node does not respond at all or does not respond within a certain acceptable time frame (Haider et al. 2011). Incorrect and erroneous response of a grid node is considered as a response failure. Response failure is further categorized into value failure and state transition failures.

- Value failure

Value failure is faced when the value of a response is wrong (Haider 2007). An unexpected or out of range value received by the grid server from a grid node for a query is an example of a value failure.

b. State transition failure

State transition failure is a problem when the messages transmitted by server are not received by clients due to network problem (Haider 2007). Moreover, failure of state transition can also be observed if a server stops sending messages due to some problem in the network.

#### Network failure

Network failure is a very serious issue, as a communication in distributed environment is impossible without a network (Das and De Sarkar 2012). Network failure can be due to site failures, link failures, configuration changes, or device failures such as routers or switches (Haider et al. 2011).

Push and pull models for the identification and detection of network failures can successfully be used (Haider 2007). Legion is a grid middleware that uses “pinging and timeout” approach to check whether a machine is alive and responding, or not (Nguyen-Tuong 2000; Grimshaw et al. 1997).

- Configuration change

Configuration change is a very important reason due to which a network is likely to fail (Haider and Ansari 2012). Participating machines of a grid belong to different networks bounded by the configuration and policies of the respective network. A change in policy or configuration may cause problems for applications using the resources of those machines (Haider et al. 2007). Due to this very fact, it is very important for a grid administrator about the implications of change in configuration on the jobs running in that environment.

According to a survey conducted by Medeiros et al. (2003) many failures are experienced in grids due to configuration related problems and solutions for the problem are mostly application dependent. Reasons identified in Medeiros et al. (2003) are that though a high-level of abstraction exists between grid components but when a problem occurs then all complex gory details are exposed that are related to configuration, middleware, hardware and software based issues.

#### Software failure

Software failure is an important class of failures in a grid environment, as the software is the most important component of the grid (Vaidyanathan and Trivedi 2001). Grid middleware is software, which requires further software, such as operating system. Moreover applications executing in the grid environment are also software (Haider et al. 2011). Software failures cannot be left unattended. Many complex issues can be experienced due to the technicality and delicacy of software.

- Operating system failure

The most fundamental type of software failure is the operating system failure. When the operating system of a grid node fails, then the execution of the application and services on that particular machine are stopped (Haider 2007). Selection of a dependable and reliable operating system is an important factor to tackle the problem of operating system failure. Historical data regarding the failures and crashes of operating system can be maintained from the perspective of proactive decisions regarding the operating system failures (Haider 2007; Haider and Ansari 2012).

b. Application and task specific failure

Application and task specific failures also belong to the software failure category. However, the reason behind application and task specific failures can be software, as well as hardware.

c. Performance failure

Performance failure is also an important class of software failures (Khan et al. 2010). Failure in the performance of software can be due to hardware (Haider et al. 2011). A slow processor or a communication link with less bandwidth can not deliver the results within an acceptable time frame and ultimately results in performance failures (Haider 2007). Bad selection of resources could also be the reason for performance failure (Haider 2007). Unhandled exceptions or exceptions generated due to unexpected inputs are all types of performance failure that ultimately are types of software failures (Vaidyanathan and Trivedi 2001).

#### Miscellaneous failure

Some of the failures identified in literature do not fall in any specific failure category and a few of them are time related and arbitrary failure (Baldoni et al. 2007) According to Baldoni et al. (2008), arbitrary failures are one of the toughest failures and is a real practical challenge due to unexpected software errors and malicious attacks. In arbitrary failures, a server is prone to generate random and arbitrary responses at arbitrary/random times.

Another type of miscellaneous failure is random failure. Task assignment to compute nodes is known as resource allocation or mapping. Mapping policies in grid environments depends upon many factors, e.g. number of available nodes, nodes characteristics and links between them. Scenarios can be developed for number of available nodes as nodes can randomly fluctuate between down and up states. SETI@Home is an example where participating nodes keep on fluctuating randomly and can join or leave the system any time due to any reason (Shestak et al. 2012). Another example of random failures could be due to malfunctioning of hardware due to harsh operating environments, e.g. temperature increase of a machine due to broken cooling fan can seriously result in performance or even malfunction of processor.

### Fault

The reason behind system or component failure is fault, and fault tolerance means that the system keeps on providing services even in the presence of faults (Haider et al. 2011). Literature survey reveals many types of faults, such as aging related faults, omission faults, response faults, and timing related faults etc., which we detail below.

#### Aging related fault

Faults that creep into the system with the passage of time are placed into the aging related faults category. The phenomenon of software based aging was reported in Garg et al. (1998), Vaidyanathan and Trivedi (2001). The observation regarding the software based aging was that once the software is started, many possible fault conditions gradually are accumulated with time leading to either performance degradation or transient failures, or both (Vaidyanathan and Trivedi 2001). Hardware faults related to aging are well known. The performance of hardware degrades as the time passes and the degradation can lead to problems, such as performance, maintainability and availability. The bathtub curve in computer architecture is well-known for identifying the reliability of a machine based on time. Klutke et al. (2003) have referenced that some products show decrease in failure rate in early life and an increase in failure in later life.

#### Omission faults

Omission faults are more prevalent in grids and arise when resources becomes unavailable (Siva Sathya and Syam Babu 2010; Garg and Singh 2011). Disk space full is considered to be omission fault as once the disk space of a hard disk completes; thereafter, further storage of data on that device cannot be stored as the storage resource is unavailable. Denial of service (DoS) is a type of omission fault where a node of the network is under the potential threat of DoS attack and will be forced to stop the services for which it is responsible.

#### Response faults

Response faults can be classified as, value faults, byzantine faults, and state transition faults. When a server responds incorrectly to a request than response faults occur (Siva Sathya and Syam Babu 2010). If some lower level system or application level fault has not been handled properly, then an individual processor or application may emit incorrect output or value, and is known as value faults (Siva Sathya and Syam Babu 2010; Haider et al. 2007). Byzantine faults take place due to failed or corrupted processors that behave arbitrarily (Coulouris et al. 2001). Byzantine faults take place when a system does not stop after a failure, and starts behaving in an unpredictable way (Siva Sathya and Syam Babu 2010). The problems faced when processes are changing their states are known as state transition faults.

#### Timing faults

Problems that occur due to synchronization between processes are known as timing faults. Timing faults arise in synchronous distributed environments where processes have strict time limitations with respect to communication or execution. Timing faults occur when the specified time limit exceeds (Avizienis et al. 2004). Timing faults are further divided into the categories of early and late faults.

When execution or communication services start too early then it is called early fault. Similarly, when communication or execution services are too late and exceed the time limit then late faults are encountered.

#### Interaction faults

Interaction faults occur when an increase number of interactions occur between a large numbers of services. Many of these services may be dynamically bounded at run time and original application developer may be unaware of such a scenario. Therefore, the result of such an increased interaction results in interaction faults (Garg and Singh 2011). A reason of interaction fault may also be due to different services supporting different protocols (Townend and Xu 2003). Timing overhead, security incompatibilities, and policy problems are the types of interaction faults.

- Policy problems and security incompatibilities

The difference in the policies of the grid nodes of different networks lead to policy issues. The problems faced by applications that interact with the grid nodes working under different policies is known to be policy problems. Security incompatibility is another type of interaction fault that could be due to policy problems.

b. Timing overhead

Application interaction with respect to timing may lead to faults. A time out in a service due to slow processor, low bandwidth, or failed link may cause problems (Townend and Xu 2003). Faults related to timing are also placed into the category of interaction faults.

#### Software faults: Heisenbugs and Bhorbugs

Hisenbugs and bhorbus are types of software failures that lead to intermittent failures. Heisenbugs cause a class of software failures that typically surface in situations where there are boundaries between various software components and are likely to appear in grids. Heisenbugs result in intermittent failures that are extremely difficult to identify through testing (Vaidyanathan and Trivedi 2001).

Bohrbugs are permanent design faults and are almost deterministic in nature. They can be identified easily and weeded out during the testing and debugging phase of the software life cycle.

#### Life cycle faults

Faults expected to occur due to different versions of applications and their toolkits. An example of versioning fault is that of a grid application developed for GT4 (globus Buyya and Murshed 2002; Klutke et al. 2003 toolkit version 4) might create problems from versioning point of view on GT3 (globus toolkit version 3).

- Service expiry fault

A particular service or resource on the grid is available for a particular time. An application that tries to use a service or resource beyond the time for which that service or resource is available would result in a life cycle type of fault known as service expiry fault.

#### Response faults: Byzantine and value faults

Response faults take place due to failed or corrupted processors that behave arbitrarily (Coulouris et al. 2001). A lower level system or application level fault that has not been handled properly may emit incorrect output. The incorrect output or value produced by application is known as value fault.

## Design goals in grid dependability

Probability of faults in a grid environment is much higher than a traditional distributed system (Nazir et al. 2009). To minimize the faults and making grids more reliable, we must strive for improving its dependability. Encountering challenges of different types as discussed in “Challenges in grid dependability” section and taking care of threats identified in “Threats to grid dependability: errors, failures and faults” section we can proceed towards dependable grids. Design goals of a dependable grid are availability, reliability, continuity, quality of service, flexibility, and adaptability.

### Availability

The most important design goal in any fault tolerant system is availability that depicts a quality responsible for providing correct services. If problems are encountered in a distributed environment, then the availability characteristic of dependability must be able to handle the problems. Reliability is another important design goal of not only in grid but in any of the fault tolerant system. Reliability portrays the willingness for the provisioning of accurate services. A system is more available if that system is reliable and vice versa. Availability and reliability are directly proportional to each other (Charoenpornwattana et al. 2008).

### Adaptability

Adaptability refers to the capability of the system that can accommodate changes and provide the specified services at the same time. An adaptive fault tolerant design improves availability and reliability of the system. Adaptable systems can respond to the changed environment and policy that otherwise can create problems and generate faults (de Lemos 2006). Many fault tolerant solutions considering adaptability have been discussed (Guimaraes et al. 2013; Sun et al. 2013; Nazir et al. 2009; de Lemos 2006; Guimaraes and de Melo 2011)

### Continuity and quality of service

Continuity and quality of service (QoS) are also related to reliability and availability. Services are dependent on the availability of the system. If a system is unavailable due to hardware or software failures, then it is obvious that the system would be unable to continue providing services. A system that is not providing or fails to provide smooth and consistent service, suffers from the problems known as continuity and quality of service.

Many of the techniques (Chan et al. 2007; Foster et al. 2003; Wei-Tek et al. 2003; Zheng and Lyu 2008; Zheng and Lyu 2009) provided are not appropriate to be used in different systems having specific performance requirements. An adaptive fault tolerance technique with QoS-aware middleware is proposed by Zheng and Lyu (2010). Zheng model is based on user collaborated QoS aware middleware that can dynamically adjust its fault tolerance configurations in order to achieve reliability and performance.

### Maintainability

Maintainability refers to the capability of performing the necessary amendments and repairs whenever required for the smooth operation and functioning of the system. If we broaden the horizon of the design goals of a dependable grid system, then security, integrity, and maintainability must also be considered.

**Fig. 6:**
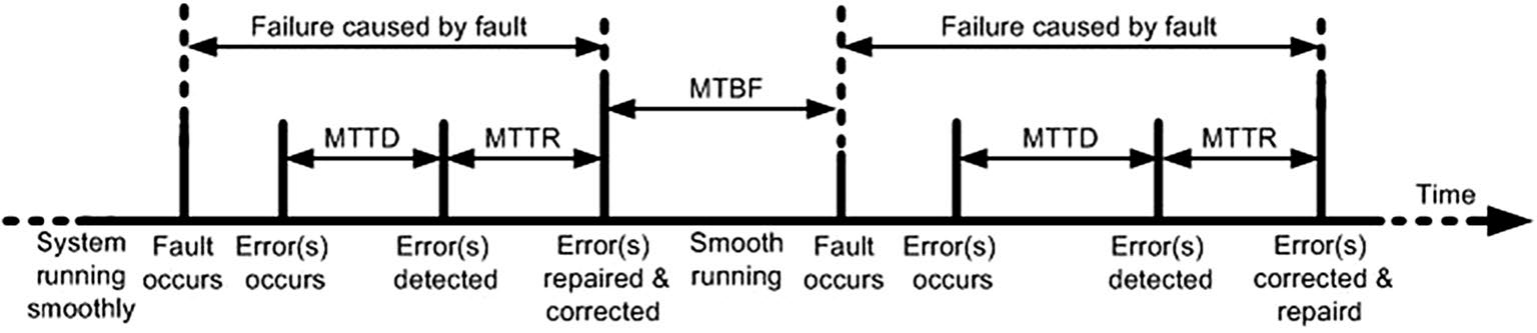
Factors required for finding availability and reliability

The design goal with respect to dependability is a concept that includes many attributes such as, availability, reliability, safety, integrity, and maintainability (Avizienis et al. 2004). In Fig. 6, we point out the parameters on which the availability and reliability of a system depends. The parameters used for determining the availability and reliability are: (a) mean time to detect (MTTD), (b) mean time to repair (MTTR), and (c) mean time between failures (MTBF). Christer Carlsson (2011) analyzed failure data, collected over several years at the Los Almos National Laboratory (LANL), where the study included the major causes of failures, the mean time between failure (MTBF), and the mean time to repair (MTTR). The researchers discovered that the average failure rates were roughly ranging from 20 to 1000 failures per year (Christer Carlsson 2011).

## Fault detection and tolerance in grid computing

As the size and sophistication of the present-day distributed systems make the occurrence of failures, the rule rather than the norm, many fault tolerant resource management techniques have been designed (Gallet et al. 2010). In Table 1 we provide a comparative analysis of various grid middleware that have fault tolerant capabilities. Literature survey reveals that grid computing paradigms in distributed environments use various fault detection and tolerance techniques, which are discussed as under:

**Table 1:** Comparison of fault detection and tolerance techniques used in grids along with their advantages and disadvantages

System	Fault detection technique	Types of faults detected	Fault tolerance technique	Advantages	Disadvantages
Globus Buyya and Murshed (2002), Klutke et al. (2003)	Heartbeat monitor	Host failure, Network failure	Resubmit the failed job	Generic failure detection	Can not handle user defined exceptions
MDS-2 Buyya and Murshed (2002), Coulouris et al. (2001)	GRRP	Task crash failure	Retry	Task crash failure detection through protocols	Can not handle user defined exceptions
Legion Alvisi and Marzullo (1998), Hussain et al. (2006)	Pinging	Task failure	Checkpoint recovery	Application level fault tolerance	Can not discern between task failure and network failure
Condor-G Townend and Xu (2003)	Polling	Host crash, Network crash	Retry on same machine	Provides security, management of jobs, and fault tolerance	Retry on same machine, can not detect task crash failure
NetSolve Buyya and Murshed (2002), de Lemos (2006)	Generic heartbeat mechanism	Host crash, task crash, and network failure	Retry on another available machine	Load balancing, heartbeat mechanism, Retry on another machine	Does not support diverse failure recovery mechanism
CoG Kits Guimaraes and de Melo (2011)	N/A	N/A	N/A	Security, Discovery of resources, and management of resources	Failure detection is hard coded, Ignores fault tolerance

Globus provides a software infrastructure that enables applications to handle distributed heterogeneous computing resources as a single virtual machine. The Globus toolkit consists of a set of components that implement basic services, such as security, resource allocation, resource management, and communications (Baker et al. 2002). Globus can be considered as a grid computing framework that offers many services for wide-area application execution to application developers. The Globus heart beat monitor (Hwang and Kesselman 2003; Stelling et al. 1999) provides a generic failure detection service designed to be incorporated into distributed system, tools, or applications. Globus enables applications to detect both host/network failure by detecting missing heartbeats. The strategy for fault tolerance used in Globus is to resubmit the failed jobs (Affaan and Ansari 2006).

Monitoring and Discovery Systems (MDS-2) in theory can support the task crash failure detection functionality through the GRRP (Gullapalli et al. 2001) notification protocol and the Grid Resource Information Service/Grid Index Information Server (GRIS/GIIS) framework. However, in case of Globus heart beat monitor, it is not straight forward to use MDS-2 to construct the failure detection services. The MDS-2 is in fact designed to develop grid information services rather than the failure detection services. Moreover user-defined exceptions cannot be detected using the MDS-2 (Hwang and Kesselman 2003; Czajkowski et al. 2001).

Legion is an object-based system developed at the University of Virginia. The software infrastructure offered by Legion ensures seamless interaction of machines in heterogeneous and geographically distributed environments. Features available in Legion are transparent scheduling, data management, fault tolerance, site autonomy, and security (Baker et al. 2002). Legion uses “pinging and timeout” mechanism to detect task failures. If a response is not received from a task within an acceptable time, then Legion assumes that the task has failed. Indeed, this pinging and timeout mechanism can detect neither the task crash failures nor user-defined exceptions, nor Legion can distinguish the pure task crash failure from the host/network failures (Nguyen-Tuong 2000; Grimshaw et al. 1997). Legion provides fault tolerance through checkpoint recovery at the application level (Medeiros et al. 2003).

Condor-G leverages software from Globus and Condor to enable users to harness multi-domain resources as if they all belong to one personal domain. Condor-G combines the inter-domain resource management protocols of the Globus toolkit. Similarly, Condor-G uses the intra-domain resource management methods of Condor. This combination allows the users to combine large collections of resource across multiple domains, providing an impression as they belong to one personal domain (Frey et al. 2002). Features offered by Condor-G are job management, resource selection, security, and fault tolerance. Condor-G (Frey et al. 2002) adopts an ad hoc failure detection mechanism because the underlying grid protocol ignores fault tolerance issues. Condor-G uses periodic polling to the generic grid server to detect certain types of failures, such as the crash of the generic grid server and host/network failures. However, Condor-G can neither detect the task crash failures nor the user-defined exceptions, as is the case in Legion. Condor-G uses retry on the same machine for fault tolerance in a grid environment (Sistla and Welch 1989). In Condor-G the idea of fault tolerance and scalability is attained by composing the system of replicable modules that can be executed on any node. Fault tolerance is provided by using “process peer fault tolerance”, when a module fails, it is restarted by one of the peers (Hussain et al. 2006).

According to Baker et al. (2002), NetSolve is a programming and runtime system for accessing high-performance libraries and resources, transparently. NetSolve (Baker et al. 2002) is a client/server application designed to solve computational science problems in a distributed environment. NetSolve is based on a loosely coupled distributed system. Performance is ensured by a load-balancing policy that enables NetSolve to use the computational resources available as efficiently as possible. Clients of NetSolve can be written in C and fortran language, and use MATLAB or the Web to interact with the server. MATLAB can be used in many areas of computer science, e.g. signal and image processing, computational biology, control systems and financial models etc.

Many MATLAB based applications for parallel programming exists. MatlabMPI (Kepner and Ahalt 2004) created by MIT Lincoln Laboratory, MultiMATLAB (Trefethen et al. 1996) by Cornell University, bcMPI by Ohio Supercomputing Center (Bliss and Kepner 2007) and pMATLAB etc. are the most notable MATLAB parallel programming applications. Furthermore, MATLAB offers specialized routines in the form of add-ons, known as “toolboxes” (Sharma and Martin 2009) along with some simple interfaces to high-performance libraries. Advantage of using NetSolve is that it ensures good performance through the load balancing policy that enables NetSolve to use the computational resources available as efficiently as possible. NetSolve uses a generic heartbeat mechanism for failure detection and uses retry on another available machine for fault tolerance (Hwang and Kesselman 2003).

The CoG Kit is a Commodity Grid toolkit that defines and implements a set of general components that map grid functionality into a commodity environment/framework (Von Laszewski et al. 2000). With the help of the CoG Kit, the application developers can exploit the advanced services of grid, such as resource management, security, and resource discovery. Similarly, CoG kit can be used for developing higher-level components in terms of familiar and powerful application development frameworks (Von Laszewski et al. 2000). CoG Kit (Hwang and Kesselman 2003) does not have failure detection mechanism and is missing the advanced features of fault tolerance, such as replication and check pointing.

### Mechanisms used for fault detection and tolerance

The following techniques can be used for detection and identification of faults in grid computing environments:

#### Push model

In push model, the components of the grid starts sending heartbeat messages at regular time intervals to a central failure detector. If failure detector does not receive a message from one or more grid components within a specified time, then failure detector assumes and considers the problem as a failure of that component (Garg and Singh 2011).

#### Pull model

For detection of faults in pull model, the failure detector keeps on sending ping requests to the grid components after specific time intervals. Ping request sent for a particular device if not received within an acceptable time frame is considered to be failure of that particular device (Garg and Singh 2011).

#### Probability based techniques

Different probability based techniques are also used to detect and identify problems that are expected in grid computing environments. Joshi et al. (2011), has used probability based approach for automating recovery of faults in distributed environments. Risks associated with service level agreements in grid environments are calculated by Carlsson and Fuller (2010), using a predictive probabilistic approach.

#### Neural network based approaches

One of the many usages of the neural networks is in the field*/*area of computer networks for diagnosing faults. Some researchers (Charoenpornwattana et al. 2008) are applying the concept for detecting and diagnosing faults in grids for improving reliability. Charoenpornwattana et al. (2008), used neural network based approach for proactive fault avoidance. Calado and da Costa (2006), used neural network based fault identification and diagnosis using fuzzy approach to achieve reliability in high performance computing environments.

#### Proactive fault tolerance

Fault tolerance c*an be* further handled intelligently by developing and adopting techniques such as maintaining the history of information about successful job completion. Faults faced/observed during the working of grid environment can also be handled proactively. The probability of resource and or node failure history can also be maintained and used later for proactive fault tolerance. Similarly, reliability of resources of grid participating nodes/machines can also be generated using algorithms resulting in timely decisions regarding fault tolerance. In proactive fault tolerance, we take decisions regarding a problem that has not yet actually occurred or observed. Although many proactive fault tolerance techniques for grids have been proposed by researchers (Nazir et al. 2012; Haider et al. 2007; Nazir et al. 2009; Vallee et al. 2008; Engelmann et al. 2009; Nagarajan et al. 2007; Litvinova et al. 2009; Benjamin Khoo and Veeravalli 2010) but still a comprehensive and acceptable proactive fault tolerance technique with respect to grid is awaited.

#### Reactive fault tolerance

Reactive fault tolerance is used in systems where job failures are considered and handled after occurrence. Most of the fault tolerant techniques are reactive in nature and many grid middleware (Hwang and Kesselman 2003; Katzela 1996; Grimshaw et al. 1997; Stelling et al. 1999; Czajkowski et al. 2001; Baker et al. 2002) are handling the issue of fault tolerance, reactively. Most of the research regarding fault tolerance in grid environments is using reactive/post-active approach that is handling faults after detection.

## Performance evaluation criteria

There are many factors that need to be considered while evaluating a good or a bad fault tolerant system. An obvious fact is that more focus and concentration on fault tolerance will be at the cost of system performance. An intelligent fault tolerant system can be designed while considering system performance in mind. Performance evaluation criteria’s in fault tolerance are identified in Table 2.

**Table 2:** Performance evaluation criteria’s

S. no.	Evaluation criteria	Recommended	In between	Not recommended
1	Time to detect errors	Early	–	Late
2	Failure probability	Low	Medium	High
3	Node selection for job execution	Intelligent	Random	Unintelligent
4	Failure detection	Proactive	Reactive	–
5	Fault detection layers	All layers	Few layers	No layer
6	Recovery time of failed node	Low	Medium	High
7	Response time after failure	Early	–	Late
8	Resource utilization	Increased	–	Decreased
9	Recovery technique	Workflow, task level	–	–
10	Job success ratio	Increased	Moderate	Decreased
11	Overall throughput	Increased	Moderate	Decreased
12	Overall ATAT	Reduced	–	Magnified
13	Errors detected	Large	Medium	Low
14	Overall AWT	Low	Medium	High
15	Transmission delay	Reduced	–	Magnified
16	Implementation	Easy	–	Difficult
17	Adaptability	Yes	–	No
18	Fault detection	Dynamic	–	Static
19	Task level FT	Checkpoint	Replication	Alternate resource
20	MTTF	Increased	–	Decreased
21	MTTR	Decreased	–	Increased
22	MTTD	Decreased	–	Increased

Performance evaluation criteria’s identified in Table 2 signify that authenticity of fault tolerant model will improve by incorporating more of its factors. It is perhaps impossible to consider all the criteria’s while designing a fault tolerant system. However, more the considered points mentioned in Table 2, better will be the designed fault tolerant system. Similarly, trying to achieve all of the defined criteria’s, and architecture will be bulky that ultimately will result in the overall reduction in performance.

## Open issues: fault tolerance in grid computing

Grid computing will keep on imposing new conceptual and technical challenges (Nazir et al. 2012). Open issues with respect to fault tolerance are to find ways to detect and handle different types of errors, failures, and faults in distributed application or middleware used in grid computing environments.

### Establish a fault detection mechanism capable of detecting faults

Various techniques can be used for detecting faults. Artificial neural network, probability, push model and pull model are the techniques that can be applied for identification of faults. Combination of two or more techniques, such as artificial neural network and probability, or any other combination can be helpful for fault detection and according to our knowledge a combination of neural network and probability based approaches have not yet been applied for fault identification in grids. Probability and neural network can also be used for treatment of faults proactively.

### Identification of the domain of the problem

The problems incurred in grids can be in the form of errors, failures, and faults. Therefore, it would be better to actually identify the problem domain. Identification of problem domain is to know whether the problem is error, failure, or fault and further getting information about the sub category of the type of problem.

### Fault repercussion analysis

After the identification of the domain of the problem, that problem must be assessed for the possible impact. For example, what type of harm or damage can that problem cause? Similarly, further investigation about the identified problem with respect to the location in the layered grid architecture and solution for improving the availability can further be helpful. Factors, such as mean time to detect and mean time to repair can then be used to check whether the proposed solution has increased availability factor or not. Solutions that improve availability are conformance of correctness and further are proof of reliability.

### Maintaining log of problems and using fault tolerance scheduling technique during resource allocation

Several research papers have shown the use of fault tolerant scheduling strategies (Latchoumy and Khader 2012; Nazir et al. 2012; Haider et al. 2007; Benjamin Khoo and Veeravalli 2010; Amoon 2012) for computational grids. An intelligent fault tolerant scheduling scheme that combines ideas from neural network, probability, and historical data gathered over a course of time can also be a smart way that can help in fault tolerance.

### Hybrid fault tolerance technique

Hybrid fault tolerance approach, such as a combination of proactive and reactive technique can also be used in grid environments. Proactive technique would actually inform about the problem before that problem is observed in the system. Moreover, if the problem is encountered then reactive techniques would be there to take over the situation.

### Prediction of failures and its impact on performance

Another important research area from fault tolerance point of view in grids and other HPC environments is of predicting failures. If an application in HPC environment is likely to finish before the predicted failure, then a proactive fault tolerant measure can be avoided, hence a possibility in improving performance (Egwutuoha 2014).

## Conclusion

In this survey we have learned that fault tolerance is an important issue that must be dealt with care, as reliability, dependability, performance, and quality of service depends upon the reliable provisioning of services.

Literature review reveals that the distributed systems are lacking a complete classification of the types of errors, failures, and faults. Every type of problem is considered and named as a fault, though it could be an error or failure too. We have created an extended classification of errors, failures and faults. To ensure reliability and dependability in a distributed application or system, all of these should be incorporated.

Different fault tolerant techniques are available for grid based environments and most of them are reactive in nature. However, most of the techniques are capable of handling only few types of errors. Very few techniques are dynamic and handle faults proactively. For fault tolerant techniques to be more efficient and precise, the emphasis must be on fault detection first, as only the correct and timely fault detection can ensure a timely and right fault tolerant mechanism.
